# Prognostic Implication and Immunological Role of PSMD2 in Lung Adenocarcinoma

**DOI:** 10.3389/fgene.2022.905581

**Published:** 2022-06-08

**Authors:** Huihui Zhao, Guojun Lu

**Affiliations:** ^1^ Department of Oncology, The Second Hospital of Nanjing, Nanjing University of Chinese Medicine, Nanjing, China; ^2^ Department of Respiratory Medicine, Nanjing Chest Hospital, Affiliated Nanjing Brain Hospital, Nanjing Medical University, Nanjing, China

**Keywords:** PSMD2, lung adenocarcinoma, prognosis, biomarker, immune infiltration

## Abstract

**Background:** Although previous studies reported that 26S proteasome non-ATPase regulatory subunit 2 (*PSMD2*) is involved in many human cancers. However, its clinical significance and function in lung adenocarcinoma remain unclear. Here, we examined the prognostic and immunological role of *PSMD2* in lung adenocarcinoma.

**Methods:** The Cancer Genome Atlas (TCGA) was conducted to analyze *PSMD2* expression and verified using UALCAN. PrognoScan and Kaplan-Meier curves were utilized to assess the effect of *PSMD2* on survival. cBioPortal database was conducted to identify the mutation characteristics of *PSMD2*. Functional enrichment was performed to determine *PSMD2*-related function. Cancer Single-cell State Atlas (CancerSEA) was used to explore the cancer functional status of *PSMD2* at single-cell resolution. *PSMD2-*related immune infiltration analysis was conducted. Tumor-Immune system interaction database (TISIDB) was performed to verify the correlation between *PSMD2* expression and tumor-infiltrating lymphocytes (TILs).

**Results:** Both mRNA and protein expression of *PSMD2* were significantly elevated in lung adenocarcinoma. High expression of *PSMD2* was significantly correlated with high T stage (*p* = 0.014), lymph node metastases (*p* < 0.001), and TNM stage *p* = 0.005). Kaplan-Meier curves indicated that high expression of *PSMD2* was correlated with poor overall survival (38.2 vs. 59.7 months, *p* < 0.001) and disease-specific survival (59.9 months vs. not available, *p* = 0.004). Multivariate analysis suggested that *PSMD2* was an independent biomarker for poor overall survival (HR 1.471, 95%CI, 1.024–2.114, *p* = 0.037). *PSMD2* had a high mutation frequency of 14% in lung adenocarcinoma. The genetic mutation of *PSMD2* was also correlated with poor overall survival, disease-specific survival, and progression-free survival in lung adenocarcinoma. Functional enrichment suggested *PSMD2* expression was involved in the cell cycle, RNA transport, and cellular senescence. CancerSEA analysis indicated *PSMD2* expression was positively correlated with cell cycle, DNA damage, and DNA repair. Immune infiltration analysis suggested that *PSMD2* expression was correlated with immune cell infiltration levels and abundance of TILs.

**Conclusion:** The upregulation of *PSMD2* is significantly correlated with poor prognosis and immune infiltration levels in lung adenocarcinoma. Our findings suggest that *PSMD2* is a potential biomarker for poor prognosis and immune therapeutic target in lung adenocarcinoma.

## Introduction

Lung cancer is still the most common cancer in China and the leading cause of cancer-related death in China and the United States of America (USA) (Lee et al., 2019; Siegel et al., 2022; Xia et al., 2022). Lung adenocarcinoma is the most common histological subtype of lung cancer and accounts for about 40% of diagnoses (Hua et al., 2020). The research to date indicates that targeted therapy and immune-checkpoint inhibitors plus chemotherapy have become standard therapy for lung adenocarcinoma and bring survival benefits (Hirsch et al., 2017; Gandhi et al., 2018; Mok et al., 2019). However, up to now, the 5-year survival rate of lung adenocarcinoma has remained a struggle at 16% (Huo et al., 2019). Furthermore, previous studies have reported that tumor-infiltrating lymphocytes (TILs) including tumor-associated macrophages and tumor-infiltrating neutrophils are correlated with the prognosis and sensitivity of chemotherapy and immunotherapy (Waniczek et al., 2017; Pan et al., 2019). As a result, it is urgent to explore the immune phenotype of lung adenocarcinoma and identify novel prognostic biomarkers and therapeutic strategies for lung adenocarcinoma.

The Proteasome 26S Subunit, Non-ATPase (*PSMD*) gene family, is composed of the *PSMD1* to *PSMD14*. Previous studies have shown that *PSMD* family genes can play important role in the progress of circulation and tumorigenesis by regulating ubiquitinated protein breakdown. *PSMD1-3* and *PSMD7* were elevated in breast cancer and correlated with poor prognosis. They can promote cell proliferation and cell cycle progression prognosis in breast cancer cell lines (Oguro et al., 2015; Okumura et al., 2018; Fararjeh et al., 2019; Zhao et al., 2020). In esophageal cancer, upregulation of *PSMD4* can reduce endoplasmic reticulum stress-induced cell apoptosis to promote tumor progression (Ma et al., 2019). It was also reported that *PSMD9* was correlated with recurrence after radiotherapy in cervical cancer and can predict radiotherapy benefits in breast cancer (Langlands et al., 2014; Köster et al., 2020). A previous study has shown that *PSMD2* was overexpressed in lung cancer and patients with higher expression of *PSMD2* were correlated with poorer prognosis (Matsuyama et al., 2011). However, the number of lung cancer patients enrolled in this study was relatively small. Moreover, *PSMD* family genes are reported to correlate with immune infiltration profiles in breast cancer (Xuan et al., 2021). Up to now, there is no studies have designed to investigate the association between *PSMD2* expression and immune cell infiltration in lung adenocarcinoma.

In this study, we first examined the expression of *PSMD2* in lung adenocarcinoma in the TCGA and UALCAN databases. Then we conducted PrognoScan and Kaplan-Meier curves to assess the correlation between *PSMD2* expression and prognosis. cBioPortal database was conducted to identify the mutation characteristics and prognostic significance of *PSMD2*. Furthermore, functional enrichment analysis, cancer functional status at single-cell resolution and immune cell infiltration analysis were also conducted. Our findings link the upregulation of *PSMD2* with the poor prognosis and propose a therapeutic immunological target for lung adenocarcinoma.

## Materials and Methods

### The Cancer Genome Atlas

TCGA (https://portal.gdc.cancer.gov/) is a landmark cancer genomics program (Tomczak et al., 2015). In the present study, the expression level of *PSMD2* in different cancer types and related clinical data in lung adenocarcinoma were downloaded from TCGA for further analysis.

### UALCAN and Clinical Proteomic Tumor Analysis Consortium Database

The UALCAN (http://ualcan.path.uab.edu/) is an online web resource to analyze cancer omics data (Chandrashekar et al., 2017). It can be used to perform protein expression analysis of the CPTAC dataset (Edwards et al., 2015). In this study, we input *PSMD2* in the “scan by gene” module of UALCAN to explore the total protein expression between primary tumor and normal tissues with the CPTAC dataset of lung adenocarcinoma.

### The Human Protein Atlas

The HPA database (https://proteinatlas.org/) contains human protein expression profiles in tumor tissues and normal tissues (Uhlén et al., 2015; Uhlen et al., 2017). In the present study, HPA was conducted to confirm the protein expression of PSMD2 in lung adenocarcinoma.

### PrognoScan

PrognoScan database (http://dna00.bio.kyutech.ac.jp/PrognoScan/index.html) is an online database for prognosis analysis (Mizuno et al., 2009). In the present study, we input *PSMD2* in the “Enter gene identifier” module of PrognoScan and validated the prognostic importance of *PSMD2* in lung adenocarcinoma with two datasets (GSE31210, GSE13213).

### cBioPortal Database

The cBioPortal for Cancer Genomics (https://www.cbioportal.org/) is a Web resource to explore, visualize, and analyze multidimensional cancer genomics data (Cerami et al., 2012; Gao et al., 2013). In this study, we utilized the cBioPortal database to assess mutation data of *PSMD2*, capture its prognostic value in altered lung adenocarcinoma patients, and acquire co-expressed genes of *PSMD2*.

### Functional Enrichment Analysis

After acquiring co-expressed genes of *PSMD2* from cBioPortal, we performed Gene Ontology (GO) and Kyoto Encyclopedia of Genes and Genomes (KEGG) pathway analysis with R package clusterProfiler to further quantify the functional annotations of these co-expressed genes (Yu et al., 2012). R package ggplot2 was adopted to visualize the results of GO and KEGG.

### Cancer Single-Cell State Atlas

CancerSEA is an online database to comprehensively decode distinct functional states of cancer cells from different cancer types at single-cell resolution (Yuan et al., 2019). In this study, the relevant data of *PSMD2* across different tumor functional states based on single-cell sequencing data were downloaded from CancerSEA and a heatmap was drawn. The t-SNE diagram was also downloaded from CancerSEA to describe the distribution of all individual cells.

### Immune Infiltration Analysis

The methods for analyzing immune infiltration were described as before (Li et al., 2021; Zhang et al., 2022). Tumor infiltration expression of 24 immune cell types was downloaded from published literature (Bindea et al., 2013) and quantified by the ssGSEA method with R package GSVA (Hänzelmann et al., 2013). Furthermore, Spearman correlation and Mann-Whitney U test were conducted to determine the correlation between low/high expression of *PSMD2* and immune cell infiltration in lung adenocarcinoma.

### Tumor-Immune System Interaction Database

TISIDB (http://cis.hku.hk/TISIDB/) is an integrated repository portal for tumor and immune system interaction (Ru et al., 2019). In the present study, TISIDB was employed to capture the relations between *PSMD2* expression and the abundance of TILs in lung adenocarcinoma. The relations between *PSMD2* expression and TILs were determined by Spearman’s test.

### Statistical Analyses

R (V 3.6.3, https://www.r-project.org/) was utilized for statistical analyses and R package ggplot2 was employed for visualization of expression differences. Paired t-test and Mann-Whitney U test were proposed to explore the expression differences between lung adenocarcinoma tissues and adjacent normal tissues. Kaplan-Meier curves and Cox regression were conducted with R package survminer and survival to assess the effect of *PSMD2* on survival. *p* < 0.05 was considered statistically significant.

## Results

### Assessment of PSMD2 mRNA Expression in Pan-Cancer Perspective and Lung Adenocarcinoma

**FIGURE 1:**
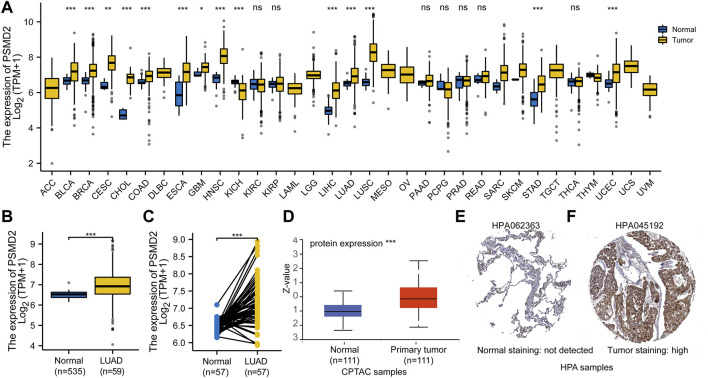
Expression pattern of *PSMD2* in Pan-cancer perspective and lung adenocarcinoma. **(A)** *PSMD2* is increased in 13 cancer types and decreased only in Kidney Chromophobe from TCGA. **(B,C)** Both the Mann-Whitney U test and paired t-test indicate mRNA expression of *PSMD2* is elevated in lung adenocarcinoma. **(D)** The protein expression of PSMD2 is upregulated in CPTAC. **(E)** The PSMD2 protein expression in normal tissue from HPA. **(F)** The PSMD2 protein expression in lung adenocarcinoma tissue from HPA. (^∗^ *p* < 0.05, ^∗^^∗^ *p* < 0.01, ^∗^^∗^^∗^ *p* < 0.001, ns, no significant).

To assess the transcription level of *PSMD2* in multiple tumors and normal samples, we conducted analyses on the TCGA database. As shown in Figure 1A, compared with normal tissue controls, the expression of *PSMD2* was upregulated in 13 cancer types and only downregulated in Kidney Chromophobe. Looking at Figure 1B, the Mann-Whitney U test indicated that the *PSMD2* mRNA expression level in lung adenocarcinoma (*n* = 535) was significantly upregulated relative to normal lung tissues (*n* = 59) (6.964 ± 0.698 vs. 6.510 ± 0.177, *p* < 0.001). As shown in Figure 1C, paired t-test analysis showed that *PSMD2* mRNA expression level in lung adenocarcinoma (*n* = 57) were significantly upregulated relative to normal lung tissues (*n* = 57) (7.156 ± 0.662 vs. 6.512 ± 0.177, *p* < 0.001). Taken together, these results suggest that mRNA expression of *PSMD2* is elevated in lung adenocarcinoma tissues.

### Assessment of PSMD2 Protein Expression in Lung Adenocarcinoma

To explore the total protein expression of PSMD2 between lung adenocarcinoma and normal tissues, we analyzed CPTAC with the UALCAN dataset. The result in Figure 1D also showed that the protein expression of PSMD2 in lung adenocarcinoma was significantly elevated than in normal tissues. Protein expression from HPA also suggested that PSMD2 in lung adenocarcinoma tissue was higher than that in normal lung tissue (Figures 1E,F). Overall, these results indicate that protein expression of PSMD2 is increased in lung adenocarcinoma tissues.

### Relationships Between PSMD2 Expression and Clinicopathological Factors of Lung Adenocarcinoma Patients

**FIGURE 2:**
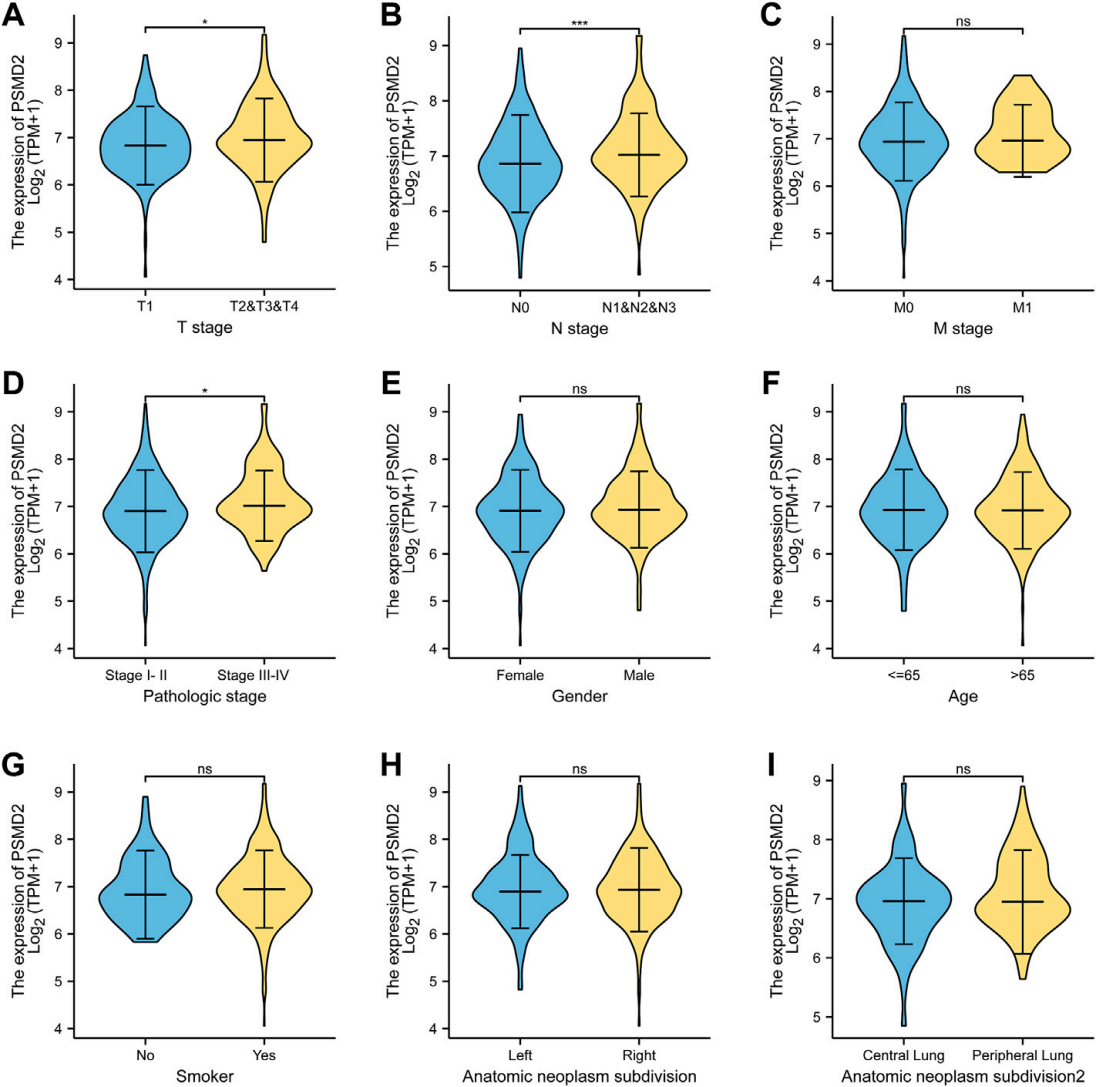
The clinical relevance of *PSMD2* expression and clinicopathological factors in lung adenocarcinoma patients. *PSMD2* mRNA expression was significantly increased with high T stage **(A)**, N stage **(B)**, and TNM stage **(D)**. No observed differences in M stage **(C)**, gender **(E)**, age **(F)**, smoke condition **(G)**, and anatomic subdivision (right vs. left, central vs. peripheral) **(H,I)**. (ns, no significance, ^∗^ *p* < 0.05, ^∗^^∗^^∗^ *p* < 0.001).

The basic clinicopathological factors of lung adenocarcinoma patients was listed in Table 1. To examine the clinical relevance of *PSMD2* expression, we conducted the expression between different clinicopathological factors of lung adenocarcinoma patients with the Mann-Whitney U test. As listed in Figure 2, the high expression of *PSMD2* was significantly correlated with T stage (T1 vs. T2-4, *p* = 0.014), N stage (N0 vs. N1-N3, *p* < 0.001), and TNM stage (Stage I-II vs. Stage III-IV, *p* = 0.005). However, *PSMD2* expression had no significant correlation with other clinicopathological factors, such as M stage (*p* = 0.401), gender (*p* = 0.174), age (*p* = 0.884), smoking condition (*p* = 0.225), and anatomic subdivision (right vs. left, *p* = 0.999; peripheral vs. central, *p* = 0.149). On the whole, these results indicate that *PSMD2* had a significant correlation with T stage, lymph node metastases, and high TNM stage.

**TABLE 1:** The basic characteristics of clinicopathological factors in lung adenocarcinoma patients.

Characteristics	Total	Low expression	High expression	*p*-value
	N (%)	N (%)	N (%)	
T stage				0.078
T1	175 (32.9)	94 (17.7)	81 (15.2)	
T2	289 (54.3)	138 (25.9)	151 (28.4)	
T3	49 (9.2)	28 (5.3)	21 (3.9)	
T4	19 (3.6)	5 (0.9)	14 (2.6)	
N stage				0.038∗
N0	348 (67.0)	186 (35.8)	162 (31.2)	
N1	95 (18.3)	40 (7.7)	55 (10.6)	
N2	74 (14.3)	29 (5.6)	45 (8.7)	
N3	2 (0.4)	1 (0.2)	1 (0.2)	
M stage				1.000
M0	361 (93.5)	174 (45.1)	187 (48.4)	
M1	25 (6.5)	12 (3.1)	13 (3.4)	
Pathologic stage				0.131
Stage I	294 (55.8)	155 (29.4)	139 (26.4)	
Stage II	123 (23.3)	62 (11.8)	61 (11.6)	
Stage III	84 (16.0)	32 (6.1)	52 (9.9)	
Stage IV	26 (4.9)	13 (2.5)	13 (2.5)	
Gender				0.759
Female	286 (53.5)	145 (27.1)	141 (26.4)	
Male	249 (46.5)	122 (22.8)	127 (23.7)	
Age				1.000
<=65	255 (49.4)	127 (24.6)	128 (24.8)	
>65	261 (50.6)	131 (25.4)	130 (25.2)	
Smoker				0.034d∗
No	75 (14.4)	46 (8.8)	29 (5.6)	
Yes	446 (85.6)	211 (40.5)	235 (45.1)	
Anatomic neoplasm subdivision				0.518
Left	205 (39.4)	107 (20.6)	98 (18.8)	
Right	315 (60.6)	154 (29.6)	161 (Zhang et al., 2022)	
Anatomic neoplasm subdivision2				0.844
Central Lung	62 (32.8)	30 (15.9)	32 (16.9)	
Peripheral Lung	127 (67.2)	58 (30.7)	69 (36.5)	

∗, p < 0.05.

### Correlation Between PSMD2 Expression and Prognosis

**FIGURE 3:**
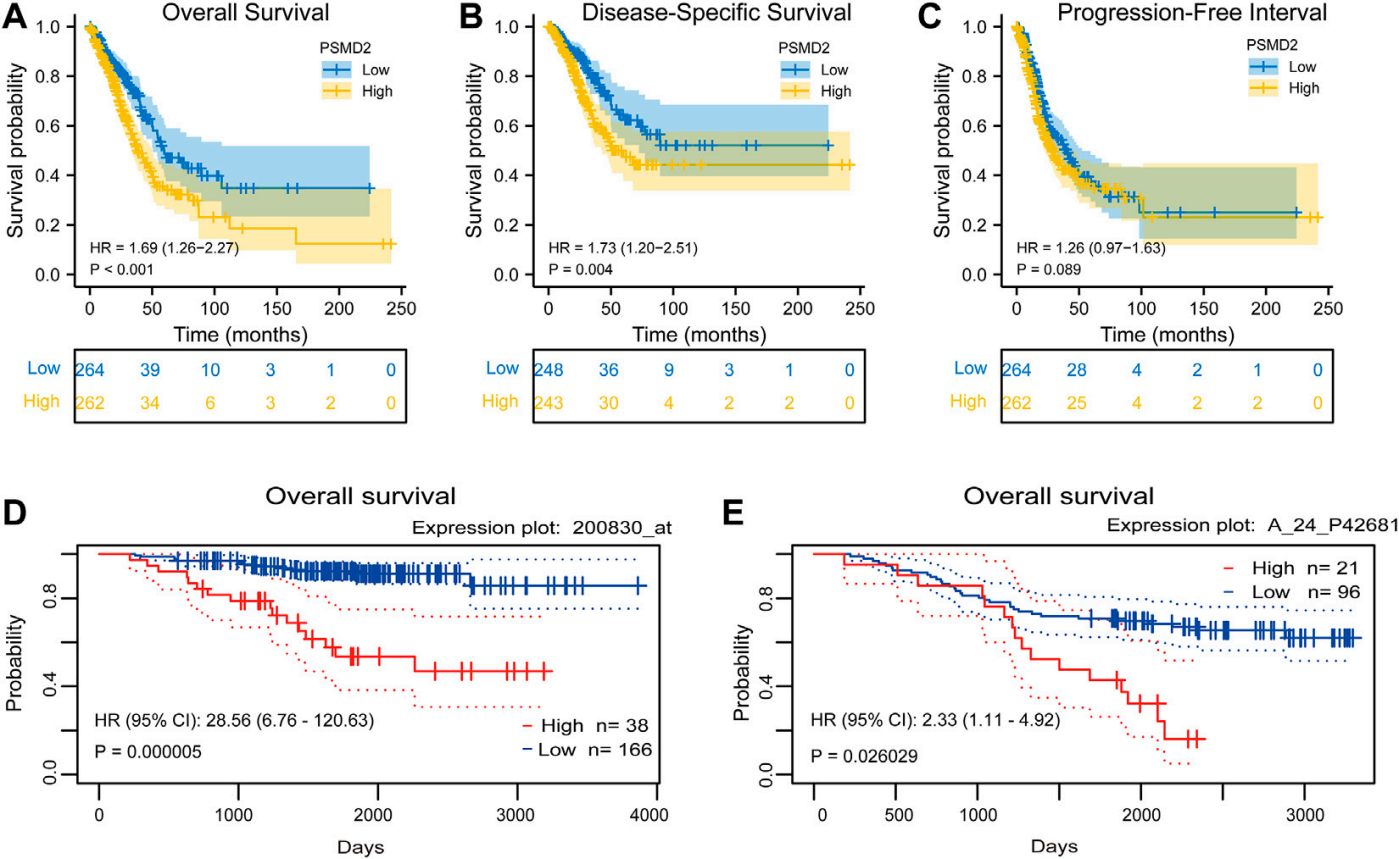
Correlation between *PSMD2* expression and prognosis in lung adenocarcinoma. **(A)** Higher *PSMD2* expression had a shorter overall survival. **(B)** Higher *PSMD2* expression had a shorter disease-specific survival. **(C)** No significant difference was observed between *PSMD2* expression and progression-free interval. **(D,E)** Two datasets (GSE31210, GSE13213) in PrognoScan suggested that high *PSMD2* expression was correlated with poor overall survival in lung adenocarcinoma.

**FIGURE 4:**
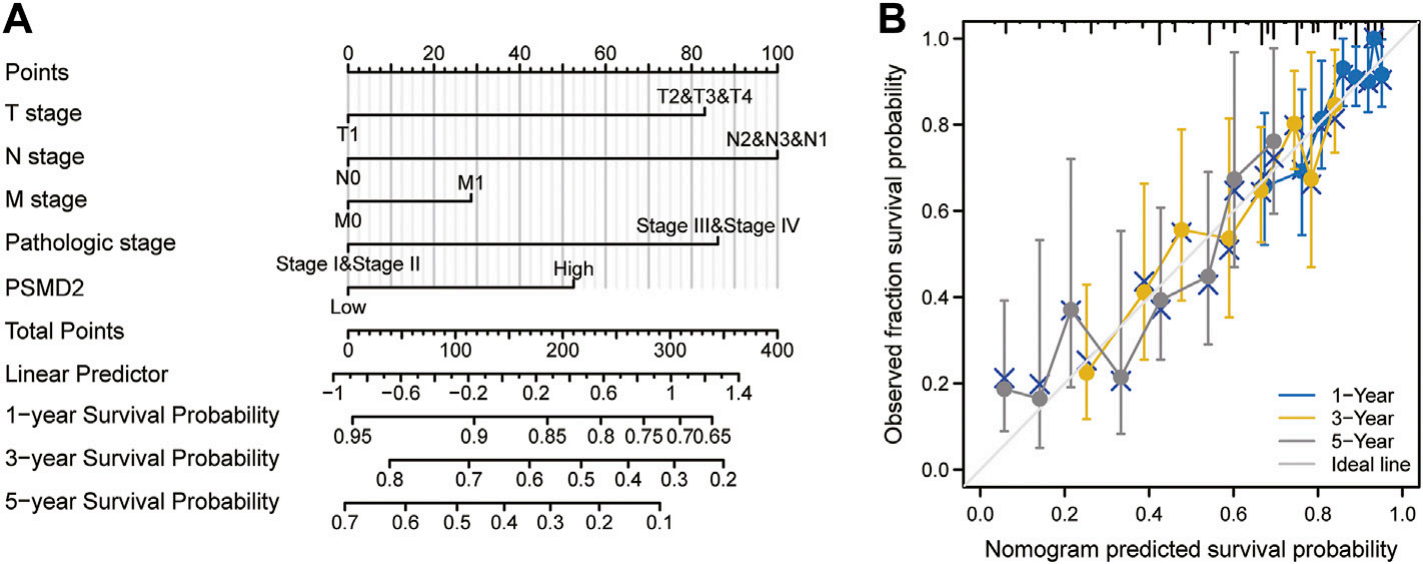
A nomogram and calibration plot for predicting the 1-, 3-, and 5-year overall survival probability of lung adenocarcinoma patients. **(A)** To predict the survival probability, first draw a vertical line from each clinical factor up to the points axis and get a point. Repeat until points for all clinical factors are decided. Sum all the points and then locate the total point on the total points axis. Draw a line straight down to the risk axis and finally obtain survival probability. **(B)** Calibration plot of the nomogram.

To investigate the correlation between *PSMD2* mRNA expression and prognosis of lung adenocarcinoma patients, Kaplan-Meier curves with R package survminer and survival were used. As can be seen in Figure 3A, the overall survival of lung adenocarcinoma patients with higher *PSMD2* expression was significantly shorter than those with lower *PSMD2* expression (38.2 vs. 59.7 months, *p* < 0.001). As shown in Figure 3B, the disease-specific survival of lung adenocarcinoma patients with higher *PSMD2* expression was also significantly shorter than those with lower *PSMD2* expression (59.9 months vs. not available, *p* = 0.004). However, as shown in Figure 3C, no significant difference was observed between higher and lower *PSMD2* expression and progression-free interval (28.8 vs. 40.1 months, *p* = 0.089). Furthermore, we validated the prognostic importance of *PSMD2* in PrognoScan. The result from two datasets in Figures 3D,E suggested that high *PSMD2* expression was correlated with poor overall survival in lung adenocarcinoma. As a result, by associating *PSMD2* and other clinical characteristics in Figures 4A,B, we established a nomogram and calibration plot for predicting the overall survival probability of lung adenocarcinoma patients.

### Prognostic Role of PSMD2 in Lung Adenocarcinoma Patients

**FIGURE 5:**
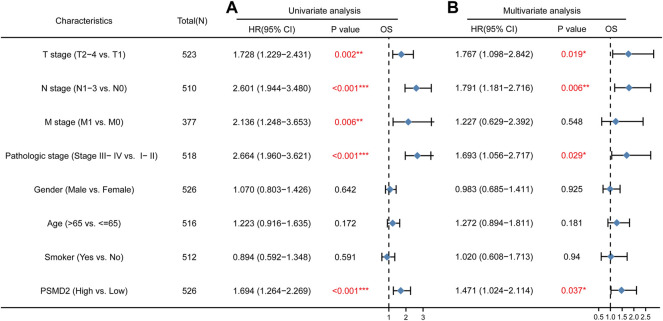
Cox regression analyses and forest plots of prognostic factors for overall survival. **(A)** Univariate Cox analysis and forest plot results of *PSMD2* for OS. **(B)** Multivariate Cox analysis and forest plot indicated *PSMD2* was an independent prognostic biomarker for OS in lung adenocarcinoma. (Red colors mean significant results. ^∗^ *p* < 0.05, ^∗^^∗^ *p* < 0.01, ^∗^^∗^^∗^ *p* < 0.001).

To further explore the prognostic role of *PSMD2* for overall survival and disease-specific survival in lung adenocarcinoma patients, we performed Cox univariate and multivariate analyses. As shown in Figure 5A, univariate analysis showed that high expression of *PSMD2* was significantly correlated with poor overall survival of lung adenocarcinoma patients (HR 1.694, 95%CI, 1.264–2.269, *p* < 0.001). Moreover, we conducted a multivariate analysis with the Cox proportional hazards model. The multivariate analysis in Figure 5B suggested that T stage (HR 1.767, 95%CI, 1.098–2.842, *p* = 0.019), N stage (HR 1.791, 95%CI, 1.181–2.716, *p* = 0.006), Pathologic stage (HR 1.693, 95%CI, 1.056–2.717, *p* = 0.029), and *PSMD2* (HR 1.471, 95%CI, 1.024–2.114, *p* = 0.037) were independent factors for overall survival in lung adenocarcinoma patients. Furthermore, as shown in Supplementary Table S1, univariate analysis showed that high expression of *PSMD2* was significantly correlated with poor disease-specific survival of lung adenocarcinoma patients (HR 1.734, 95%CI, 1.198–2.512, *p* = 0.004). However, the multivariate analysis suggested *PSMD2* (HR 1.615, 95%CI, 0.998–2.614, *p* = 0.051) was not an independent factor for disease-specific survival in lung adenocarcinoma. Our data indicate that *PSMD2* is an independent factor for overall survival in lung adenocarcinoma patients.

### Genetic Mutation of PSMD2 was Correlated With Poor Prognosis

**FIGURE 6:**
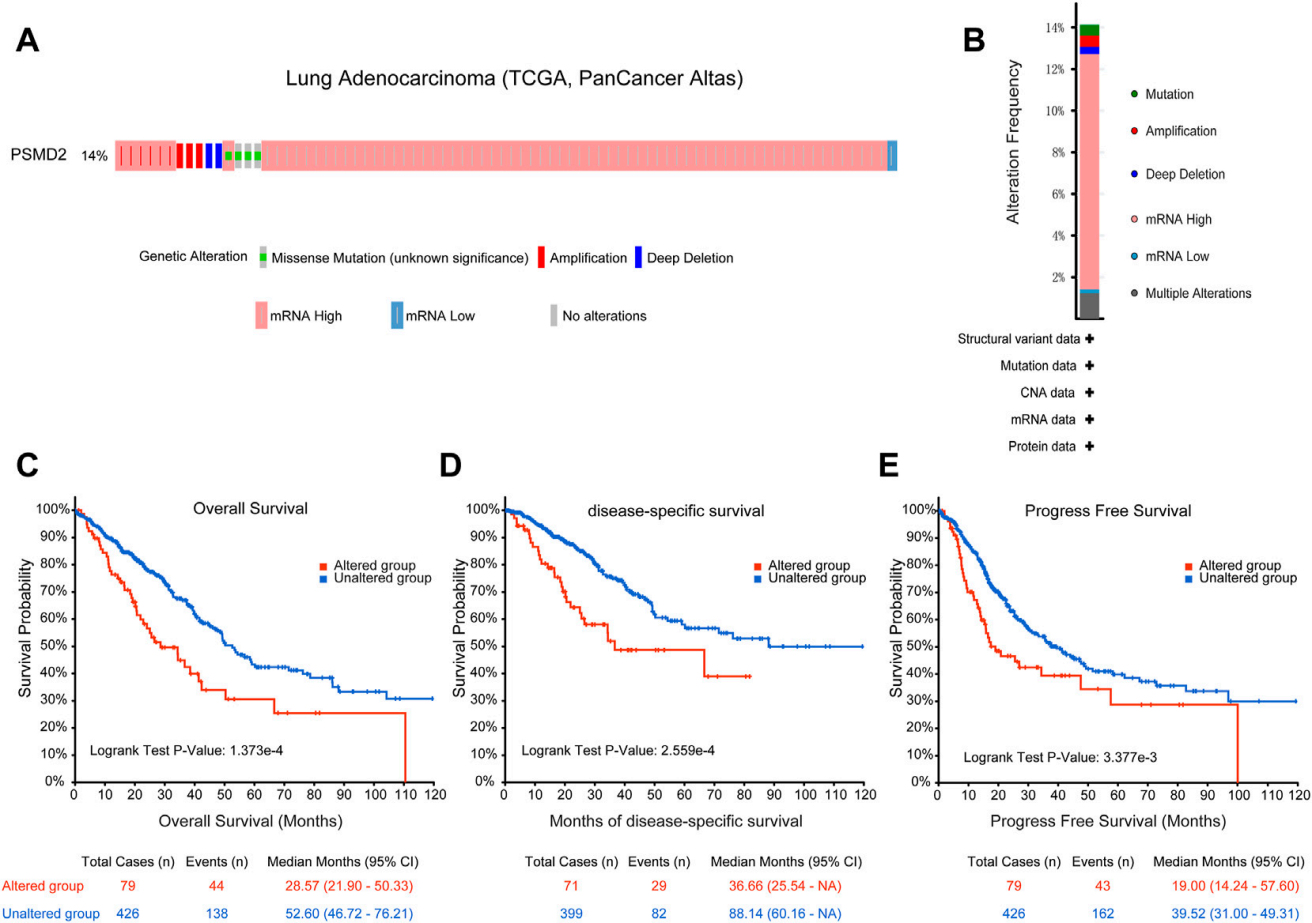
Genomic mutation of *PSMD2* in lung adenocarcinoma. **(A)** OncoPrint presented the different mutation types and proportions of *PSMD2*. **(B)** Cancer types summary showed the alteration frequency. **(C–E)** Genomic mutation of *PSMD2* was correlated with poor overall survival, disease-specific survival, and progression-free survival.

To identify the mutation characteristics of *PSMD2* and its prognostic value in altered lung adenocarcinoma patients, we utilized an analysis on the cBioPortal database. It can be seen from Figure 6A that *PSMD2* had a high mutation frequency of 14% in lung adenocarcinoma (TCGA, PanCancer Atlas). The main alteration frequency of *PSMD2* was mRNA upregulation and amplification (Figure 6B). Moreover, compared with the unaltered group, the result of Kaplan-Meier curves found that the altered group was associated with poor prognosis in overall survival (Figure 6C, 28.57 vs. 52.60 months, *p* = 1.737e-4), disease-specific survival (Figure 6D, 36.66 vs. 88.14 months, *p* = 2.559e-4), and progression-free survival (Figure 6E, 19.00 vs. 39.52 months, *p* = 3.377e-4). Overall, these results suggest that genetic mutation of *PSMD2* was correlated with poor prognosis in lung adenocarcinoma.

### Functional Enrichment Analysis of Co-expressed Genes of PSMD2

**FIGURE 7:**
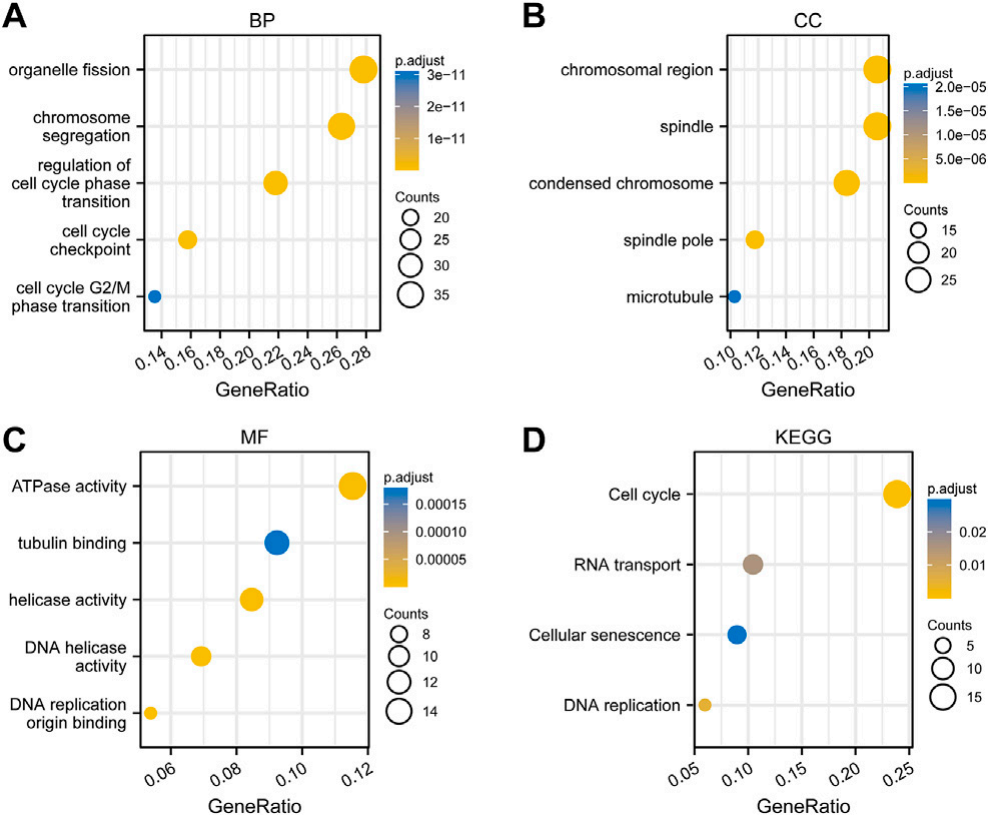
Functional enrichment analysis of co-expressed genes of *PSMD2*. **(A)** Biological progress of organelle fission, chromosome segregation, and regulation of cell cycle phase transition. **(B)** Structural constituents in the condensed chromosome, spindle, and chromosomal region. **(C)** The molecular function of ATPase activity, tubulin binding, and DNA helicase activity. **(D)** KEGG pathway analysis indicated enrichment function in the cell cycle, RNA transport, and cellular senescence.

To perform GO and KEGG analysis, we first downloaded co-expressed genes of *PSMD2* from cBioPortal. As shown in Supplementary Table S2, with |cor Spearman| > 0.5 and *p* < 0.05, *PSMD2* had 139 co-expressed genes, including 137 positive and two negative co-expressed genes. R package clusterProfiler was utilized for GO and KEGG analysis. As shown in Figure 7A, GO analysis revealed that these co-expressed genes of *PSMD2* were involved in the biological progress of organelle fission, chromosome segregation, and regulation of cell cycle phase transition. They acted as structural constituents in the condensed chromosome, spindle, and chromosomal region (Figure 7B), and played an important part in the molecular function of ATPase activity, tubulin binding, and DNA helicase activity (Figure 7C). KEGG pathway analysis in Figure 7D indicated enrichment function in cell cycle, RNA transport, and cellular senescence.

### The Correlation Between PSMD2 Expression and Cancer Functional States

**FIGURE 8:**
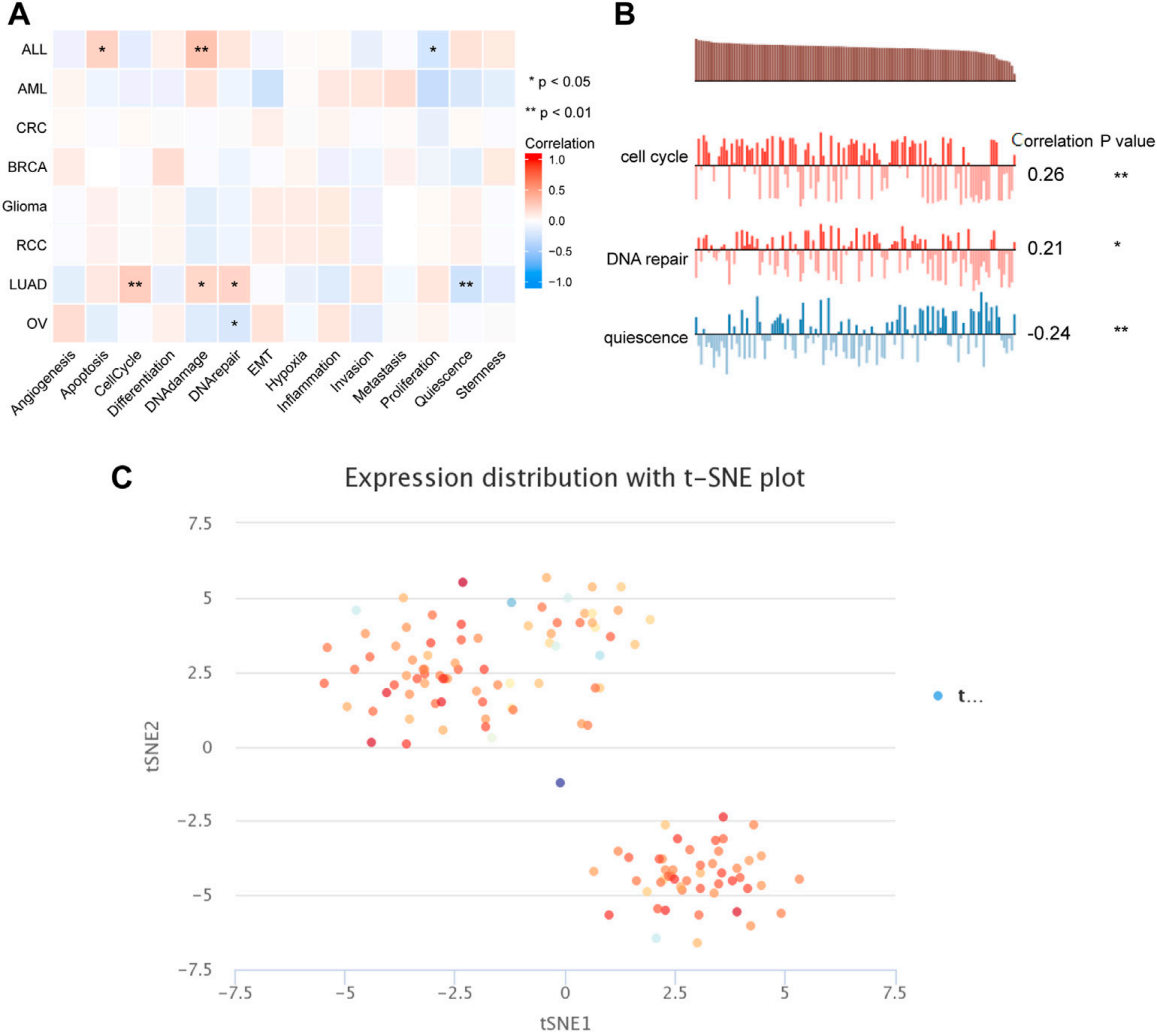
The correlation between *PSMD2* expression and cancer functional states. **(A)** A heatmap indicated the relationship between *PSMD2* expression and cancer functional states from CancerSEA. **(B)** *PSMD2* expression was correlated with cell cycle, DNA repair, and quiescence in lung adenocarcinoma. **(C)** The t-SNE diagram described the *PSMD2* expression profile in single cells of lung adenocarcinoma.

To capture the expression of *PSMD2* at single-cell resolution and its correlation with cancer functional states, we conducted an analysis on CancerSEA. The results listed in Figure 8A suggested that *PSMD2* expression was significantly positively correlated with angiogenesis and DNA damage, while negatively correlated with proliferation in acute lymphoblastic leukemia (ALL). *PSMD2* expression was positively correlated with cell cycle, DNA damage, and DNA repair, while negatively correlated with quiescence in lung adenocarcinoma (Figure 8B). The t-SNE diagram in Figure 8C described the *PSMD2* expression profile in single cells of lung adenocarcinoma.

### PSMD2 Was Correlated With Immune Infiltration in Lung Adenocarcinoma

**FIGURE 9:**
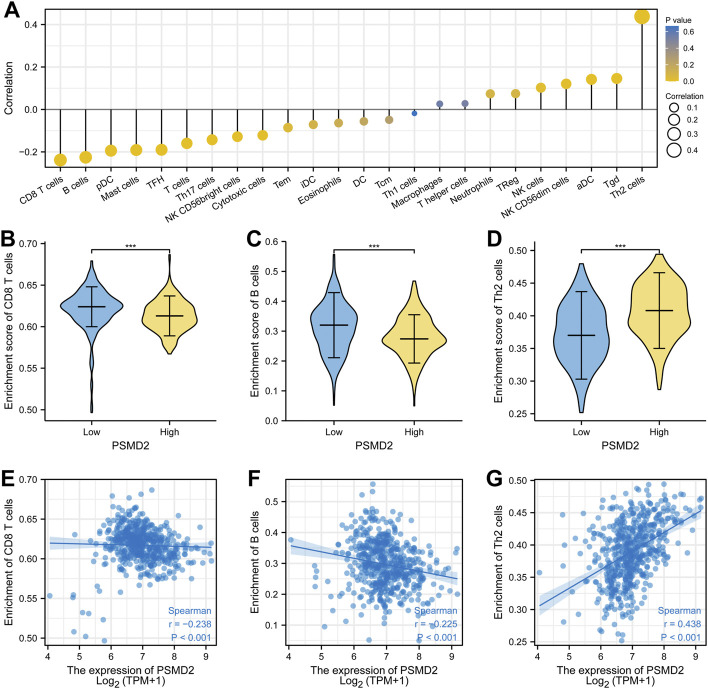
Spearman correlation between *PSMD2* expression and immune cell infiltration. **(A)** The correlation between *PSMD2* expression and the relative abundances of 24 immune cells. **(B–D)** Mann-Whitney U test indicated there were differences in immune cells (CD8 T cells, B cells, Th2 cells) between high/low expression of *PSMD2*. **(E–G)** Scatter diagrams for correlation between immune cells (CD8 T cells, B cells, Th2 cells) and *PSMD2* expression.

To analyze the correlation between the expression of *PSMD2* and immune cell infiltration, we conducted ssGSEA method with R package GSVA. The results of Spearman correlation analysis were listed in Figures 9A–G and Table 2. It was apparent that *PSMD2* expression was negatively correlated with the immune cell infiltration levels of CD8 T cells (r = −0.238, *p* < 0.001), B cells (r = −0.225, *p* < 0.001), pDC (r = −0.194, *p* < 0.001), mast cells (r = −0.191, *p* < 0.001), TFH (r = −0.190, *p* < 0.001), Tcells (r = −0.160, *p* < 0.001), Th17 cells (r = −0.143, *p* < 0.001), NK CD56bright cells (r = −0.128, *p* < 0.001), cytotoxic cells (r = −0.122, *p* < 0.001), and positively correlated with the immune cell infiltration levels of Th2 cells (r = 0.438, *p* < 0.001), Tgd (r = 0.146, *p* < 0.001), aDC (r = 0.142, *p* < 0.001), NK CD56dim cells (r = 0.121, *p* < 0.001), and NK cells (r = 0.142, *p* < 0.001). On the whole, these results suggest that *PSMD2* may regulate the level of tumor-infiltrating immune cells to affect lung adenocarcinoma progression.

**TABLE 2:** The correlation between PSMD2 expression and immune infiltration.

Gene	Immune cells	Pearson correlation		Spearman correlation	
		R	*p-*value	R	*p-*value
PSMD2	aDC	0.174	<0.001***	0.142	0.001**
PSMD2	B cells	−0.189	<0.001***	−0.225	<0.001***
PSMD2	CD8 T cells	−0.032	0.466	−0.238	<0.001***
PSMD2	Cytotoxic cells	−0.057	0.185	−0.122	0.005**
PSMD2	DC	0.035	0.423	−0.056	0.194
PSMD2	Eosinophils	−0.018	0.684	−0.064	0.140
PSMD2	iDC	0.007	0.868	−0.071	0.100
PSMD2	Macrophages	0.038	0.384	0.026	0.547
PSMD2	Mast cells	−0.143	<0.001***	−0.191	<0.001***
PSMD2	Neutrophils	0.125	0.004**	0.074	0.088
PSMD2	NK CD56bright cells	0.015	0.733	−0.128	0.003**
PSMD2	NK CD56dim cells	0.178	<0.001***	0.121	0.005**
PSMD2	NK cells	0.275	<0.001***	0.102	0.018*
PSMD2	pDC	−0.142	0.001**	−0.194	<0.001***
PSMD2	T cells	−0.139	0.001**	−0.160	<0.001***
PSMD2	T helper cells	−0.019	0.664	0.028	0.518
PSMD2	Tcm	−0.171	<0.001***	−0.049	0.258
PSMD2	Tem	−0.103	0.018*	−0.086	0.048*
PSMD2	TFH	−0.121	0.005**	−0.190	<0.001***
PSMD2	Tgd	0.172	<0.001***	0.146	<0.001***
PSMD2	Th1 cells	0.014	0.742	−0.019	0.668
PSMD2	Th17 cells	−0.158	<0.001***	−0.143	<0.001***
PSMD2	Th2 cells	0.431	<0.001***	0.438	<0.001***
PSMD2	TReg	0.152	<0.001***	0.075	0.085

*, p < 0.05, **, p < 0.01, ***, p < 0.001.

### The Relations Between PSMD2 Expression and Abundance of Tumor-Infiltrating Lymphocytes in Tumor-Immune system interaction database

**FIGURE 10:**
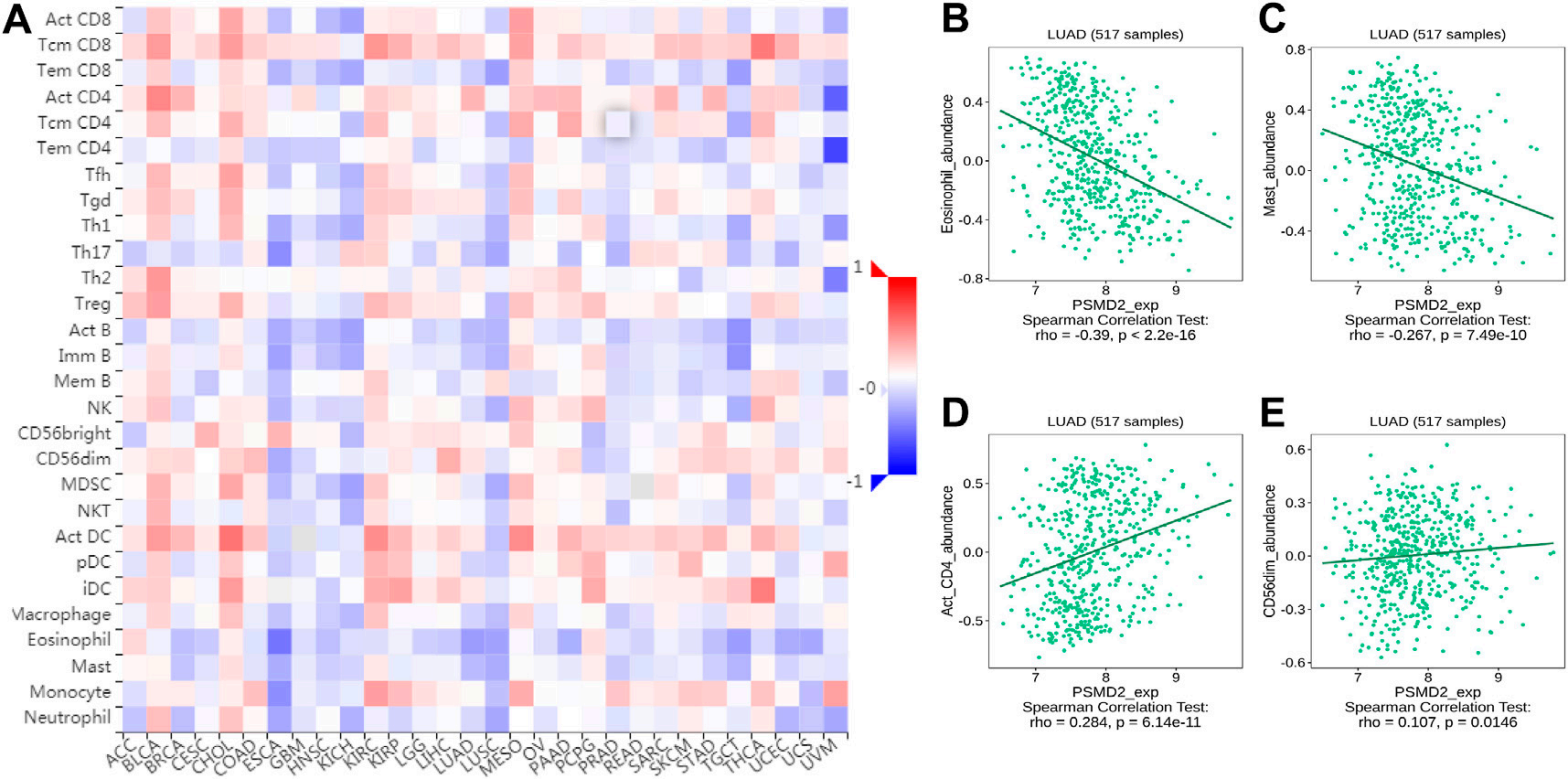
The relations between *PSMD2* expression and abundance of TILs in TISIDB. **(A)** Relations between *PSMD2* expression and 28 types of TILs across human cancers. **(B–E)** Scatter diagrams for correlation between *PSMD2* expression and abundance of eosinophil cells, mast cells, activated CD4 cells, and CD56dim cells.

To further confirm the relations between *PSMD2* expression and TILs, we performed an analysis on TISIDB. As shown in Figure 10A, we found that *PSMD2* expression was negatively correlated with abundance of eosinophil cells (r = −0.39, *p* < 2.2e-16), mast cells (r = −0.267, *p* = 7.49e-10), activated B cells (r = −0.262, *p* = 1.79e-09), Th17 (r = −0.216, *p* = 7.23e-07), immature B cells (r = −0.204, *p* = 3.03e-06), tem CT8 T cells (r = −0.191, *p* = 1.28e-05), pDC (r = −0.169, *p* = 1.19e-04), macrophage cells (r = −0.163, *p* = 2.09e-04), and neutrophil cells (r = −0.152, *p* = 5.34e-04), NK cells (r = −0.136, *p* = 1.89e-03), TFH (r = −0.117, *p* = 7.59e-03), Th1 (r = −0.106, *p* = 0.016). *PSMD2* expression was negatively correlated with abundance of activated CD4 cells (r = 0.284, *p* = 6.14e-11), CD56dim cells (r = 0.107, *p* = 1.46e-02). The results were similar to Figure 9. Scatter diagrams for correlation between *PSMD2* expression and abundance of eosinophil cells, mast cells, activated CD4 cells and CD56dim cells were listed in Figures 10B–E. Taken together, these results indicate that *PSMD2* expression is correlated with most TILs in lung adenocarcinoma, further suggesting that *PSMD2* may play an important role in the lung adenocarcinoma microenvironment.

## Discussion

In this study, we reported that the expression level of *PSMD2* is significantly elevated in lung adenocarcinoma and its upregulation is a reliable predictor of high T stage, lymph node metastases, and high TNM stage. In light of the PrognoScan database, Kaplan-Meier curves, and multivariate Cox analysis, our results confirmed that high expression of *PSMD2* is correlated with poor prognosis and *PSMD2* is an independent prognostic biomarker for overall survival of lung adenocarcinoma patients. Our results further indicated that the genetic mutation of *PSMD2* was also correlated with poor overall survival, disease-specific survival, and progression-free survival in lung adenocarcinoma. The genetic mutation of *PSMD2* was also correlated with poor overall survival, disease-specific survival, and progression-free survival in lung adenocarcinoma. Moreover, immune infiltration analysis suggested that *PSMD2* expression had a significant correlation with the level of tumor-infiltrating immune cells, further suggesting a specific role for *PSMD2* in the immunological interactions in lung adenocarcinoma.

As a member of The *PSMD* gene family, *PSMD2* has been characterized as an important non-ATPase regulatory subunit of the 19S proteasome (Li et al., 2019). Previous studies elucidated that proteasome can induce ubiquitination and degradation of protein, which in turn play an important role in the development of cell proliferation, apoptosis, and cell cycle (Chesnel et al., 2006). Functioning as a component of the proteasome, *PSMD2* was reported to play a vital role in tumor progression. In colorectal cancer, *PSMD2* can facilitate the degradation of diverse Ras-related GTPase and then inhibit cell proliferation and affect the expression of cell-cycle protein *via* blocking NF-kappaB signaling (Ying et al., 2022). In breast cancer, a paper from Li *et al.* reported that *PSMD2* can interact with p21 and p27, mediate the degradation of their ubiquitin-proteasome, and then promote cell proliferation and cell cycle progression in breast cancer (Oguro et al., 2015). In hepatocellular carcinoma, *PSMD2* can modulate cellular lipid metabolism to regulate HepG2 cell proliferation *via* p38-JNK and AKT signaling (Tan et al., 2019). In the present study, our results showed that the mRNA and protein expression of *PSMD2* is elevated in lung adenocarcinoma tissues. Moreover, we analyzed the correlation between *PSMD2* expression and the clinicopathological factors of lung adenocarcinoma patients. The current study suggests that high expression of *PSMD2* is significantly correlated with high T stage, N stage, and TNM stage. Given our results, it is likely that *PSMD2* is involved in tumorigenesis and metastasis of lung adenocarcinoma. Furthermore, both functional enrichment analysis and CancerSEA results indicate *PSMD2* expression is correlated with cell cycle, further suggesting that *PSMD2* can regulate cell cycle to promote cancer progression. However, this should be tested in other experiments.

Previous studies demonstrated that upregulation of *PSMD2* is correlated with poor prognosis in many cancers. A paper from established that patients with high *PSMD2* expression have poor overall survival and progression-free survival in bladder urothelial carcinoma Salah Fararjeh et al. (2021). Based on the result of univariate and multivariate analysis, *PSMD2* has been identified as an independent prognostic biomarker for overall survival in bladder urothelial carcinoma (Salah Fararjeh et al., 2021). In breast cancer, reported that upregulation of *PSMD2* is associated with shorter overall survival and distant-metastasis-free survival, further suggesting that *PSMD2* could act as a factor for an unfavorable prognosis in breast cancer Li et al. (2018). Our findings on the prognostic value of *PSMD2* in lung adenocarcinoma are consistent with these reports. In this study, our results indicate that lung adenocarcinoma patients with high *PSMD2* expression have poor overall survival and progression-free survival. Univariate and multivariate analysis found that *PSMD2* is an independent prognostic factor for overall survival in lung adenocarcinoma patients. Furthermore, mutation characteristics of *PSMD2* from the cBioPortal database also suggest that the altered group is associated with poor prognosis in overall survival, disease-specific survival, and progression-free survival in lung adenocarcinoma. Based on our data, we conclude that *PSMD2* is a potential biomarker for poor prognosis in lung adenocarcinoma.

Many studies have described that tumor-infiltrating immune cells are the representative cellular components and play an important part in host antitumor immune responses (Oguro et al., 2015). There is also some evidence that infiltrating immune cells is closely associated with the efficacy of immunotherapy (Zhang et al., 2020). The findings indicate that compared with high immune cell infiltration, patients with low immune cell infiltration levels may appear poor outcomes to conventional therapy in some solid tumors (Tobin et al., 2019). A paper from reported that the expression of *PSMD* member genes including *PSMD2* is related to markers of six tumor-infiltrating immune cell types in breast cancer Xuan et al. (2021). In this study, we analyzed the correlation between the expression of *PSMD2* and immune cell infiltration. By ssGSEA method, we found that *PSMD2* expression was negatively correlated with the immune cell infiltration levels of CD8 T cells, B cells, pDC, mast cells, TFH, T cells, Th17 cells, NK CD56bright cells, and cytotoxic cells. Moreover, *PSMD2* expression is positively correlated with the immune cell infiltration levels of Th2 cells, Tgd, aDC, NK CD56dim cells, and NK cells. To further confirm the relations between *PSMD2* expression and TILs, we performed an analysis on TISIDB. The results from TISIDB were similar to the ssGSEA method. Our findings suggest that *PSMD2* expression is correlated with immune cell infiltration and raise the possibility that *PSMD2* can be a potential immunotherapy target in lung adenocarcinoma. However, this hypothesis should be tested with further research.

In conclusion, our findings showed that *PSMD2* expression is significantly elevated in lung adenocarcinoma and its upregulation is a reliable predictor of high T stage, lymph node metastases, and high TNM stage. Our results confirmed that high expression of *PSMD2* is correlated with poor prognosis and *PSMD2* is an independent prognostic biomarker for lung adenocarcinoma patients. Moreover, *PSMD2* expression had a significant correlation with the level of tumor-infiltrating immune cells.

## Data Availability

The original contributions presented in the study are included in the article/Supplementary Material, further inquiries can be directed to the corresponding author.
