# Supplementary material for: Prognostic Implication and Immunological Role of PSMD2 in Lung Adenocarcinoma
Source: Front Genet. 2022 Jun 8;13:905581. doi: 10.3389/fgene.2022.905581 (PMC9214243; doi:10.3389/fgene.2022.905581)
Supplement: Supplementary file 1 [file Table1.DOCX]

**Supplementary Table 1.** Univariate and multivariate analyses of prognostic variables for disease specific survival

| Characteristics | Total(N) | Univariate analysis | |  | | Multivariate analysis | |
| --- | --- | --- | --- | --- | --- | --- | --- |
|  |  | Hazard ratio (95% CI) | P value | |  | Hazard ratio (95% CI) | P value |
| T stage (T2-4 vs. T1) | 488 | 1.850 (1.195-2.865) | 0.006** | |  | 1.909 (1.046-3.485) | 0.035* |
| N stage (N1-3 vs. N0) | 475 | 2.703 (1.873-3.900) | <0.001*** | |  | 1.815 (1.043-3.158) | 0.035* |
| M stage (M1 vs. M0) | 344 | 2.455 (1.269-4.749) | 0.008** | |  | 2.179 (0.913-5.202) | 0.079 |
| Pathologic stage (Stage III-IV vs. I-II) | 483 | 2.436 (1.645-3.605) | <0.001*** | |  | 1.179 (0.607-2.290) | 0.626 |
| Gender (Male vs. Female) | 491 | 0.989 (0.687-1.424) | 0.954 | |  | 0.771 (0.478-1.243) | 0.286 |
| Age (>65 vs. <=65) | 481 | 1.013 (0.701-1.464) | 0.944 | |  | 1.087 (0.679-1.739) | 0.728 |
| Smoker (Yes vs. No) | 477 | 1.040 (0.602-1.796) | 0.889 | |  | 1.306 (0.646-2.643) | 0.458 |
| PSMD2 (High vs. Low) | 481 | 1.734 (1.198-2.512) | 0.004** | |  | 1.615 (0.998-2.614) | 0.051 |

*, *P* < 0.05; **, *P* < 0.01, ***, *P* < 0.001

Abbreviations: CI, confidence interval
