# Supplementary material for: Prognostic Implication and Immunological Role of PSMD2 in Lung Adenocarcinoma
Source: Front Genet. 2022 Jun 8;13:905581. doi: 10.3389/fgene.2022.905581 (PMC9214243; doi:10.3389/fgene.2022.905581)
Supplement: Supplementary file 2 [file Table2.DOCX]

**Supplementary Table 3** The co-expressed genes of ALDOA

| **Correlated Gene** | **Cytoband** | **Spearman's Correlation** | **p-Value** | **q-Value** |
| --- | --- | --- | --- | --- |
| SENP2 | 3q27.2 | 0.737 | 1.21e-88 | 2.43e-84 |
| FBXO45 | 3q29 | 0.718 | 6.65e-82 | 6.64e-78 |
| EIF4G1 | 3q27.1 | 0.704 | 1.49e-77 | 9.95e-74 |
| LSG1 | 3q29 | 0.694 | 2.16e-74 | 1.08e-70 |
| OPA1 | 3q29 | 0.677 | 1.23e-69 | 4.93e-66 |
| FXR1 | 3q26.33 | 0.661 | 2.85e-65 | 9.48e-62 |
| RFC4 | 3q27.3 | 0.660 | 3.96e-65 | 1.13e-61 |
| AP2M1 | 3q27.1 | 0.659 | 1.01e-64 | 2.53e-61 |
| ABCF3 | 3q27.1 | 0.655 | 7.91e-64 | 1.75e-60 |
| MCM2 | 3q21.3 | 0.653 | 2.51e-63 | 5.01e-60 |
| PCYT1A | 3q29 | 0.637 | 2.07e-59 | 3.75e-56 |
| DCUN1D1 | 3q26.33 | 0.637 | 2.26e-59 | 3.76e-56 |
| WDR53 | 3q29 | 0.630 | 1.13e-57 | 1.74e-54 |
| ACTL6A | 3q26.33 | 0.629 | 2.03e-57 | 2.90e-54 |
| GMPS | 3q25.31 | 0.627 | 3.69e-57 | 4.91e-54 |
| PAK2 | 3q29 | 0.620 | 1.86e-55 | 2.32e-52 |
| RSRC1 | 3q25.32 | 0.615 | 2.36e-54 | 2.78e-51 |
| CKAP2L | 2q14.1 | 0.599 | 5.40e-51 | 6.00e-48 |
| CIP2A | 3q13.13 | 0.597 | 1.43e-50 | 1.50e-47 |
| KPNA1 | 3q21.1 | 0.592 | 1.58e-49 | 1.57e-46 |
| NAA50 | 3q13.31 | 0.592 | 1.66e-49 | 1.58e-46 |
| ISY1 | 3q21.3 | 0.590 | 3.03e-49 | 2.75e-46 |
| KPNA4 | 3q25.33 | 0.587 | 1.22e-48 | 1.06e-45 |
| FYTTD1 | 3q29 | 0.585 | 3.33e-48 | 2.77e-45 |
| PRR11 | 17q22 | 0.585 | 4.09e-48 | 3.27e-45 |
| PSMD11 | 17q11.2 | 0.584 | 6.62e-48 | 5.09e-45 |
| TPX2 | 20q11.21 | 0.583 | 9.42e-48 | 6.97e-45 |
| PARL | 3q27.1 | 0.575 | 2.70e-46 | 1.88e-43 |
| RPL39L | 3q27.3 | 0.575 | 2.73e-46 | 1.88e-43 |
| NCBP2 | 3q29 | 0.575 | 3.66e-46 | 2.44e-43 |
| KIF23 | 15q23 | 0.573 | 9.20e-46 | 5.93e-43 |
| DNAJB11 | 3q27.3 | 0.572 | 9.84e-46 | 6.14e-43 |
| KPNA2 | 17q24.2 | 0.571 | 1.65e-45 | 9.99e-43 |
| MFN1 | 3q26.33 | 0.571 | 2.17e-45 | 1.28e-42 |
| CDCA8 | 1p34.3 | 0.568 | 7.97e-45 | 4.55e-42 |
| TOPBP1 | 3q22.1 | 0.564 | 4.48e-44 | 2.49e-41 |
| GFM1 | 3q25.32 | 0.562 | 8.71e-44 | 4.60e-41 |
| NDC1 | 1p32.3 | 0.562 | 8.75e-44 | 4.60e-41 |
| KIF2C | 1p34.1 | 0.561 | 1.11e-43 | 5.67e-41 |
| CLSPN | 1p34.3 | 0.561 | 1.49e-43 | 7.42e-41 |
| LRRC42 | 1p32.3 | 0.559 | 2.92e-43 | 1.42e-40 |
| ALG3 | 3q27.1 | 0.559 | 3.07e-43 | 1.46e-40 |
| ORC1 | 1p32.3 | 0.559 | 3.14e-43 | 1.46e-40 |
| ECT2 | 3q26.31 | 0.558 | 4.25e-43 | 1.93e-40 |
| NCAPH | 2q11.2 | 0.558 | 4.50e-43 | 2.00e-40 |
| KIF4A | Xq13.1 | 0.556 | 8.67e-43 | 3.77e-40 |
| DHX36 | 3q25.2 | 0.556 | 9.97e-43 | 4.24e-40 |
| ANLN | 7p14.2 | 0.556 | 1.05e-42 | 4.36e-40 |
| PIGX | 3q29 | 0.555 | 1.31e-42 | 5.33e-40 |
| BUB1 | 2q13 | 0.555 | 1.54e-42 | 6.13e-40 |
| DEPDC1 | 1p31.3 | 0.554 | 1.97e-42 | 7.70e-40 |
| MYBL2 | 20q13.12 | 0.554 | 2.11e-42 | 8.10e-40 |
| DVL3 | 3q27.1 | 0.553 | 2.92e-42 | 1.10e-39 |
| CDC6 | 17q21.2 | 0.553 | 3.56e-42 | 1.32e-39 |
| STIL | 1p33 | 0.552 | 4.25e-42 | 1.55e-39 |
| UMPS | 3q21.2 | 0.551 | 6.74e-42 | 2.40e-39 |
| MRPL47 | 3q26.33 | 0.551 | 8.08e-42 | 2.83e-39 |
| AUNIP | 1p36.11 | 0.550 | 1.01e-41 | 3.47e-39 |
| EXO1 | 1q43 | 0.550 | 1.23e-41 | 4.17e-39 |
| TTK | 6q14.1 | 0.549 | 1.56e-41 | 5.20e-39 |
| ATP13A3 | 3q29 | 0.549 | 1.78e-41 | 5.81e-39 |
| CDCA5 | 11q13.1 | 0.547 | 4.69e-41 | 1.51e-38 |
| PLK1 | 16p12.2 | 0.546 | 5.72e-41 | 1.81e-38 |
| CCNB2 | 15q22.2 | 0.545 | 8.19e-41 | 2.56e-38 |
| YEATS2 | 3q27.1 | 0.544 | 1.45e-40 | 4.46e-38 |
| PRC1 | 15q26.1 | 0.542 | 3.00e-40 | 9.04e-38 |
| CCNA2 | 4q27 | 0.542 | 3.03e-40 | 9.04e-38 |
| CENPO | 2p23.3 | 0.541 | 3.85e-40 | 1.13e-37 |
| DLGAP5 | 14q22.3 | 0.541 | 4.07e-40 | 1.18e-37 |
| SHCBP1 | 16q11.2 | 0.541 | 4.46e-40 | 1.27e-37 |
| CENPA | 2p23.3 | 0.540 | 5.38e-40 | 1.51e-37 |
| CDC20 | 1p34.2 | 0.540 | 7.17e-40 | 1.99e-37 |
| RACGAP1 | 12q13.12 | 0.539 | 1.05e-39 | 2.86e-37 |
| CENPI | Xq22.1 | 0.538 | 1.15e-39 | 3.07e-37 |
| MCM6 | 2q21.3 | 0.538 | 1.15e-39 | 3.07e-37 |
| SGO1 | 3p24.3 | 0.537 | 1.66e-39 | 4.36e-37 |
| EIF2B5 | 3q27.1 | 0.536 | 3.01e-39 | 7.80e-37 |
| MAD2L1 | 4q27 | 0.534 | 6.15e-39 | 1.57e-36 |
| MCM10 | 10p13 | 0.533 | 9.73e-39 | 2.46e-36 |
| TMEM41A | 3q27.2 | 0.533 | 1.01e-38 | 2.52e-36 |
| ECE2 | 3q27.1 | 0.530 | 2.44e-38 | 6.01e-36 |
| TBCCD1 | 3q27.3 | 0.530 | 3.05e-38 | 7.42e-36 |
| FOXM1 | 12p13.33 | 0.529 | 4.79e-38 | 1.15e-35 |
| RAD54L | 1p34.1 | 0.528 | 5.79e-38 | 1.38e-35 |
| ZNF639 | 3q26.33 | 0.527 | 7.64e-38 | 1.80e-35 |
| BUB1B | 15q15.1 | 0.525 | 1.55e-37 | 3.61e-35 |
| MRPL3 | 3q22.1 | 0.525 | 1.63e-37 | 3.74e-35 |
| MCM4 | 8q11.21 | 0.525 | 2.04e-37 | 4.60e-35 |
| DIAPH3 | 13q21.2 | 0.525 | 2.05e-37 | 4.60e-35 |
| CMAHP | 6p22.3 | -0.523 | 3.40e-37 | 7.54e-35 |
| RAD51 | 15q15.1 | 0.522 | 5.94e-37 | 1.30e-34 |
| TOP2A | 17q21.2 | 0.521 | 7.06e-37 | 1.53e-34 |
| NCAPG | 4p15.31 | 0.521 | 7.38e-37 | 1.58e-34 |
| ERCC6L | Xq13.1 | 0.520 | 1.00e-36 | 2.13e-34 |
| COPB2 | 3q23 | 0.519 | 1.46e-36 | 3.06e-34 |
| NCAPG2 | 7q36.3 | 0.519 | 1.56e-36 | 3.24e-34 |
| NUP155 | 5p13.2 | 0.517 | 2.80e-36 | 5.78e-34 |
| ESPL1 | 12q13.13 | 0.517 | 3.23e-36 | 6.58e-34 |
| SLC2A1 | 1p34.2 | 0.517 | 3.79e-36 | 7.64e-34 |
| CHAF1B | 21q22.12-q22.13 | 0.517 | 3.87e-36 | 7.74e-34 |
| KIFC1 | 6p21.32 | 0.516 | 4.03e-36 | 7.97e-34 |
| CDC45 | 22q11.21 | 0.516 | 4.36e-36 | 8.54e-34 |
| SENP5 | 3q29 | 0.516 | 4.99e-36 | 9.68e-34 |
| SKA1 | 18q21.1 | 0.515 | 6.67e-36 | 1.28e-33 |
| GTSE1 | 22q13.31 | 0.515 | 7.13e-36 | 1.36e-33 |
| FANCI | 15q26.1 | 0.515 | 7.85e-36 | 1.48e-33 |
| MELK | 9p13.2 | 0.513 | 1.28e-35 | 2.39e-33 |
| MTFR2 | 6q23.3 | 0.513 | 1.53e-35 | 2.83e-33 |
| SPC25 | 2q31.1 | 0.512 | 1.81e-35 | 3.31e-33 |
| CTSV | 9q22.33 | 0.512 | 2.02e-35 | 3.66e-33 |
| DTL | 1q32.3 | 0.512 | 2.09e-35 | 3.76e-33 |
| NEK2 | 1q32.3 | 0.511 | 2.65e-35 | 4.73e-33 |
| GPN1 | 2p23.3 | 0.511 | 3.15e-35 | 5.57e-33 |
| HMCES | 3q21.3 | 0.510 | 4.68e-35 | 8.21e-33 |
| WDR76 | 15q15.3 | 0.510 | 4.75e-35 | 8.25e-33 |
| KIF11 | 10q23.33 | 0.509 | 5.26e-35 | 9.06e-33 |
| RAB7A | 3q21.3 | 0.508 | 7.30e-35 | 1.25e-32 |
| CCNB1 | 5q13.2 | 0.508 | 7.45e-35 | 1.26e-32 |
| TUBA1B | 12q13.12 | 0.508 | 7.61e-35 | 1.28e-32 |
| WDHD1 | 14q22.2-q22.3 | 0.506 | 1.44e-34 | 2.40e-32 |
| HJURP | 2q37.1 | 0.505 | 2.22e-34 | 3.67e-32 |
| SPATA6L | 9p24.2-p24.1 | -0.505 | 2.33e-34 | 3.81e-32 |
| PIK3CA | 3q26.32 | 0.505 | 2.36e-34 | 3.84e-32 |
| FAM83D | 20q11.23 | 0.505 | 2.48e-34 | 3.99e-32 |
| RAD51AP1 | 12p13.32 | 0.505 | 2.64e-34 | 4.22e-32 |
| FAAP24 | 19q13.11 | 0.504 | 2.98e-34 | 4.72e-32 |
| NDC80 | 18p11.32 | 0.504 | 3.19e-34 | 5.02e-32 |
| SPC24 | 19p13.2 | 0.504 | 3.24e-34 | 5.06e-32 |
| BRIP1 | 17q23.2 | 0.504 | 3.31e-34 | 5.13e-32 |
| HLTF | 3q24 | 0.504 | 3.62e-34 | 5.57e-32 |
| SGO2 | 2q33.1 | 0.503 | 3.91e-34 | 5.96e-32 |
| TUBA1C | 12q13.12 | 0.503 | 4.87e-34 | 7.37e-32 |
| TRIP13 | 5p15.33 | 0.503 | 5.27e-34 | 7.91e-32 |
| TICRR | 15q26.1 | 0.502 | 5.62e-34 | 8.38e-32 |
| CEP55 | 10q23.33 | 0.502 | 6.14e-34 | 9.09e-32 |
| BRCA1 | 17q21.31 | 0.502 | 6.60e-34 | 9.69e-32 |
| DDIAS | 11q14.1 | 0.502 | 7.61e-34 | 1.11e-31 |
| PSMD12 | 17q24.2 | 0.501 | 7.99e-34 | 1.16e-31 |
| PDCD10 | 3q26.1 | 0.501 | 9.45e-34 | 1.36e-31 |
